# Personalized Modeling for Prediction with Decision-Path Models

**DOI:** 10.1371/journal.pone.0131022

**Published:** 2015-06-22

**Authors:** Shyam Visweswaran, Antonio Ferreira, Guilherme A. Ribeiro, Alexandre C. Oliveira, Gregory F. Cooper

**Affiliations:** 1 Department of Biomedical Informatics, University of Pittsburgh, Pittsburgh, Pennsylvania, United States of America; 2 The Intelligent Systems Program, University of Pittsburgh, Pittsburgh, Pennsylvania, United States of America; 3 Department of Informatics, Federal University of Maranhão—UFMA, Sao Luis, MA, Brazil; New York University School of Medicine, UNITED STATES

## Abstract

Deriving predictive models in medicine typically relies on a population approach where a single model is developed from a dataset of individuals. In this paper we describe and evaluate a personalized approach in which we construct a new type of decision tree model called decision-path model that takes advantage of the particular features of a given person of interest. We introduce three personalized methods that derive personalized decision-path models. We compared the performance of these methods to that of Classification And Regression Tree (CART) that is a population decision tree to predict seven different outcomes in five medical datasets. Two of the three personalized methods performed statistically significantly better on area under the ROC curve (AUC) and Brier skill score compared to CART. The personalized approach of learning decision path models is a new approach for predictive modeling that can perform better than a population approach.

## Introduction

The use of predictive models for risk assessment, diagnosis, prognosis, and effect of therapy has the potential to improve clinical decision-making, which in turn can improve outcomes in individuals. The typical approach for modeling clinical outcomes is to derive a single predictive model from a dataset of individuals in whom the outcomes are known, and then apply the model to predict outcomes for future individuals [1]. We call such a model a *population model* since it is intended to be applied to an entire population of future individuals and is optimized to have good predictive performance *on average* on all members of the population. An alternative approach is motivated by the observation that in the current person for whom we want to predict an outcome, typically the features are known and can provide additional information that is useful in deriving a predictive model. We distinguish between a variable and a feature; a feature is a variable that has been assigned a value. As an example, if *V* is a variable that denotes sex and can take the values *female* and *male*, then *V* = *female* and *V* = *male* are two distinct features, and a person will have only one of the two features for *V*. An example of the alternative approach for modeling clinical outcomes is a *personalized model* that is specialized to the features of the current person, and it is optimized to predict especially well for that person [1–4]. Both population and personalized approaches use a dataset of individuals, and both approaches use features of the current person. The two approaches differ in how the features are used; while the population approach uses the features to *apply* a model to predict an outcome in the current person, the personalized approach uses the features to both *derive* the model and *apply* it to predict the outcome.

**Fig 1:**
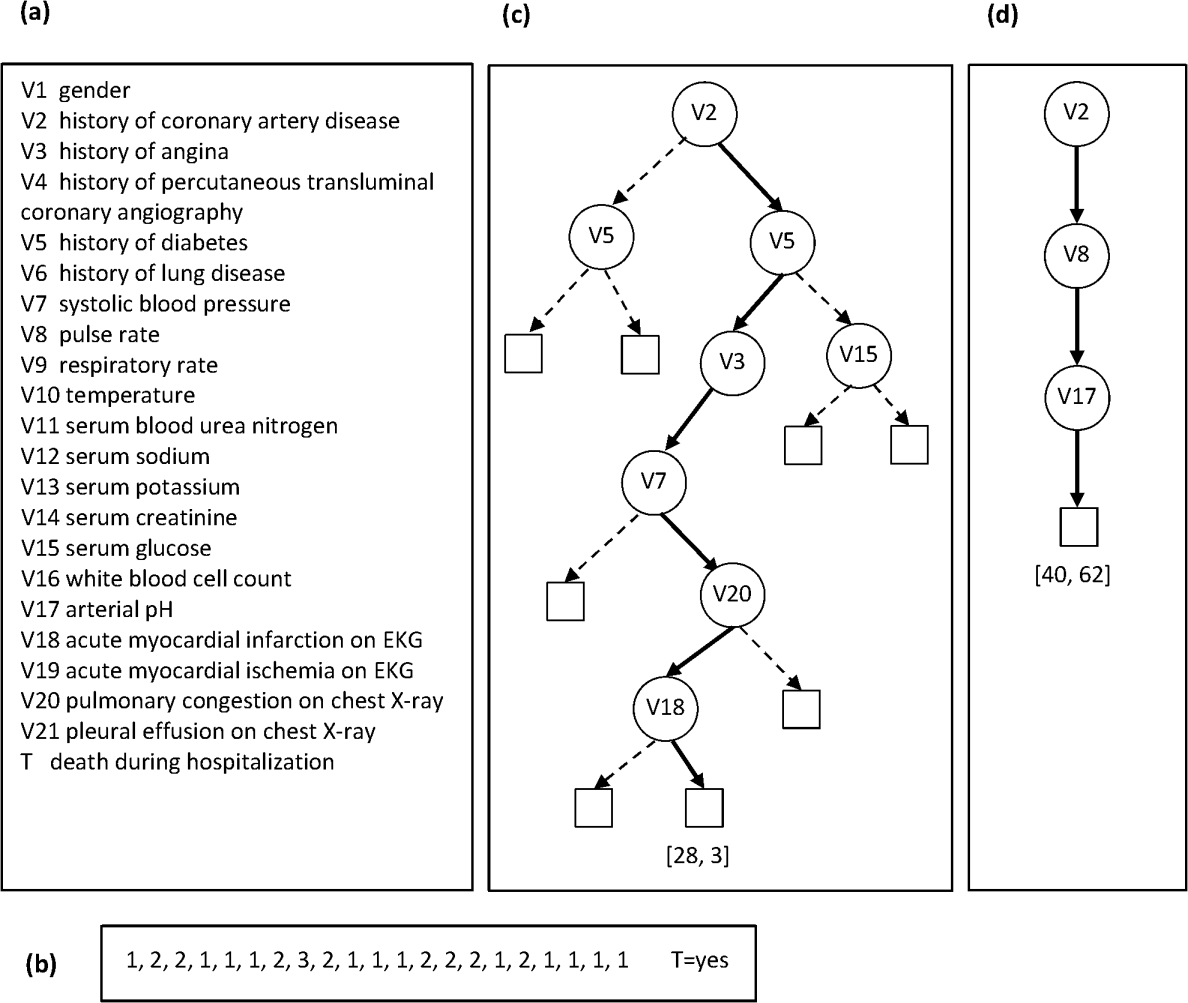
An example population decision tree and a personalized decision path. Panel (a) gives the names of the 21 variables and panel (b) gives their values for a test (current) patient whose outcome we want to predict. Panel (c) shows a population decision tree (derived by CART) and the path used for performing inference, and panel (d) shows a personalized decision path (derived by the DP-BAY method that is described later) for the patient in (b).

In some circumstances, it is possible to derive the same type of predictive model using either a population or a personalized approach. For example, a *probabilistic decision tree* is a population model that is popular in biomedicine [5, 6]. A tree consists of interior nodes that represent variables and leaf nodes that represent predictions for the target variable (such as a clinical outcome). A *path* from the root node of the tree to a leaf node represents conjunctions of features (variable values), and the leaf node at the end of the path represents a probability distribution over the target variable (see Fig 1). Decision trees can also be derived using a personalized approach; such a tree is a single path consisting of a conjunction of features that are present in the current person and a leaf node at the end of the path that represents a probability distribution over the target variable. We call such a model a *decision-path* model, which is derived specifically for the current person.

Our hypothesis is that the personalized decision-path models have better predictive performance compared to population decision trees because the former take advantage of the features in the current person while building the model. However, personalized models can suffer from overfitting when model parameters are estimated from samples whose members are similar to the current person, since such samples may be small. Thus, personalized modeling methods often incorporate mechanisms to prevent such overfitting. Personalized methods that derive decision-path models have been shown to outperform population methods that derive decision trees on several evaluation measures [7]. In this paper, we develop and evaluate the performance of three personalized methods that derive decision-path models that improve upon the methods that are described in [7]. These methods are personalized due to the following two characteristics: 1) the decision-path models contain features specific to the current person, and 2) a personalized search strategy is used for identifying good models. We compare the performance of the personalized methods with that of Classification and Regression Trees (CART), which is a commonly used population method for deriving decision trees. In this paper, we focus on decision tree models because they are widely used in medicine [6, 8–10]. These models have advantages over other types of predictive models in that they are easy to interpret due to an intuitive graphical representation, and they can be translated to a set of IF-THEN rules for improved interpretability or for implementation in clinical decision support systems.

## Background

In this section, we briefly describe decision trees that are derived using population approaches and describe lazy decision trees that use a personalized approach.

### Decision trees

A probabilistic decision tree consists of a tree *structure* that is composed of interior nodes that represent variables and a set of *parameters* in the leaf nodes that encode probability distributions of the target variable. The tree partitions the variable space into a number of non-overlapping regions and each region corresponds to a path in the tree. A decision tree can be viewed as a set of probabilistic rules, wherein each rule corresponds to a distinct path in the tree. More specifically, the antecedent of the rule is a set of conjuncts where each conjunct *V* = *v* corresponds to the variable *V* in an interior node and the branch with the value *v* emanating from the node, and the consequent of the rule corresponds to the probability distribution in the leaf at the end of the path that corresponds to the rule.

To apply a decision tree (i.e., to perform inference) to a test person, a path is identified such that the features in the path match the features in the test person. Then, the target probability distribution is estimated from the known outcomes of individuals in the training dataset whose features match the features in the identified path. Of note, each path in the tree defines a subpopulation in the training dataset, and each member of the training dataset is assigned to exactly one path in the tree.

#### Methods for deriving decision trees

A decision tree method derives both the structure and the parameters from a training dataset, and typically uses a top-down, greedy approach called recursive splitting to optimize a criterion like error or entropy. The approach is top-down because the method begins at the top of the tree and then successively splits the variable space; each split results in new branches that are created further down on the tree. The approach is greedy because at each step, the best split is made at that particular step, rather than looking ahead and picking a split that will lead to a better tree in some future step.

Typically, the top-down approach results in a tree that is too complex and likely overfits the data leading to poor predictive performance. To obtain a parsimonious model, the tree growing phase is followed with a tree pruning phase when branches are pruned from the bottom of the tree to obtain a final smaller tree.

Decision tree methods differ in the criterion that is used for selecting the variable to be split at each step. Common criteria include the Gini index (used by CART), information gain (used by ID3 and C4.5), and misclassification error [11].

### Lazy decision trees

Given a training dataset and a test person not in the training dataset, the lazy decision tree method derives a decision path that is guided by the features of the test person. The method uses a greedy approach to successively select features to add to the path; at each step a feature is chosen such that it optimizes the selected splitting criterion. After a feature is selected only those individuals in the training dataset who match the features in the path derived so far are retained to derive the remaining part of the path.

Friedman et al. [12] were the first to implement a lazy decision tree method called LazyDT and used information gain as the splitting criterion. When compared to several decision tree methods including ID3, C4.5 without pruning, and C4.5 with pruning, LazyDT had higher predictive accuracy overall and performed substantially better than ID3 and C4.5 without pruning. The main limitation of LazyDT is that it does not perform pruning and hence it may overfit.

## Decision Path Methods

In this section, we first provide a small illustrative example to contrast the population tree model with the personalized decision-path model, and later describe the decision path (DP) methods that we have developed and evaluated. The DP methods derive a decision-path model for each member of a test dataset and use it to predict an outcome of interest. As currently implemented, these methods handle only discrete predictor variables and target (outcome) variables.

We developed and implemented three DP methods that differ in the splitting criterion that is used: 1) the DP-BAY method uses a *Bayesian score* as the splitting criterion, 2) the DP-IG method uses *information gain* as the criterion, and 3) the DP-AUC method uses *area under the ROC curve* (*AUC*) as the criterion. The first two DP methods optimize a Bayesian measure and an information-theoretic measure, respectively, and the last method optimizes the AUC evaluation criterion. For the experiments described in this paper, we only consider datasets with binary target variables; however, the splitting criterions based on the Bayesian score, information gain, and the AUC are readily applicable with no (for the Bayesian score and information gain) or little (for the AUC) modification to discrete target variables with more than two states.

### Population decision tree versus personalized decision path

Fig 1 gives an example of a CART model and a personalized decision path (using the DP-BAY method that is described later) that were derived from a heart failure dataset that is described in the Experimental Methods section (see heart failure-d in Table 1). Briefly, this dataset contains 21 predictor variables and a binary outcome variable denoting *death* during hospitalization (that takes the values *no* and *yes*). Panel (a) gives the names of the 21 variables and panel (b) gives their values for a test (current) patient whose outcome we want to predict. Panel (c) shows the decision tree derived by CART and the path used for performing inference, and panel (d) shows the decision path derived by the DP-BAY method. For the test patient, the CART model yields the counts (28, 3) for the outcome values [*no*, *yes*], while decision-path model yields the counts (40, 62). Using a probability threshold of 0.5, the CART model wrongly predicts that the outcome is *no* for death, while the decision-path model correctly predicts that the outcome is *yes* for death. Moreover, the decision-path is a simpler model with a smaller number of features (three) compared to the path in the tree that has a larger (seven) number of features.

**Table 1:** Brief descriptions of the datasets.

Dataset	# Variables (cnt + dsc = total	Target variable	Sample size	Positive outcome count (percent)
pneumonia	38 + 120 = 158	dire outcome	2,287	261 (11.4%)
sepsis-d	7 + 14 = 21	death	1,673	189 (11.3%)
sepsis-s	7 + 14 = 21	severe sepsis	1,673	478 (28.6%)
heart failure-d	11 + 10 = 21	death	11,178	500 (4.5%)
heart failure-c	11 + 10 = 21	complications incld. death	11,178	1,255 (11.2%)
HIT	50 + 9 = 59	HIT	549	76 (13.8%)
Alzheimer	0 + 100 = 100	Alzheimer’s disease	1,411	861 (61.0%)

The # Variables column gives the number of continuous (cnt) and discrete (dsc) predictor variables, as well as the total number of variables (which excludes the target variable). The target variables are all binary and denote the presence or absence of a clinical outcome.

Of note, for some patients the path identified by the personalized method also exists in the CART model; in this case the personalized path and the population tree produce identical predictions. However, in other patients, the personalized path does not exist in the population tree (as in the example shown in Fig 1), and, the two methods, in general, produce different predictions.

### The DP-BAY method

We describe the DP-BAY method in detail and then briefly summarize how the other two DP methods differ from it. First, we describe the decision-path model, and then we provide details of the search strategy employed by DP-BAY to identify high scoring models.

#### Decision-path model

A decision-path model *M* consists of a pair (*S*, ***θ***) where *S* is a path and ***θ*** are the parameters of the probability distribution over target *T*. The path *S* consists of a conjunction (union) of *J* features *V*_1_ = *v*_1_ ∧ *V*_2_ = *v*_2_ ∧ *V*_*j*_ = *v*_*j*_… ∧*V*_*J*_ = *v*_*J*_ where the variable list ***V***_*S*_ = (*V*_1_, *V*_2_, …, *V*_*j*_, …, *V*_*J*_) contains a subset of all variables ***V*** in the domain and the list ***v***_*S*_ = (*v*_1_,*v*_2_,…, *v*_*j*_,…, *v*_*J*_) contains the corresponding variable-values in the test person. The parameter list ***θ*** = (*θ*_1_, *θ*_2_,…, *θ*_*k*_,…, *θ*_*K*_) denotes the *K* parameters of the multinomial distribution *P*(*T* | ***V***_*S*_ = ***v***_*S*_) over the target variable *T* conditioned on ***V***_*S*_ = ***v***_*S*_ and the values of the parameters are estimated from the individuals in the training dataset that satisfy ***V***_*S*_ = ***v***_*S*_. To control for overfitting, we use a Bayesian estimator called the BDeu measure for calculating the probability parameters [13]; the parameter *θ*_*k*_ is given by

$$\theta_{k}\equiv P\left(T=k|V_{S}=v_{S}\right)=\frac{\alpha_{k}+N_{k}}{\alpha +N},$$ (1)

where (1) *N* is the number of individuals in the training dataset *D* that satisfy ***V***_*S*_ = ***v***_*S*_ and *N*_*k*_ is the number of individuals that satisfy ***V***_*S*_ = ***v***_*S*_ and *T* = *k*, (2) *N* = ∑_*k*_ *N*_*k*_, (3) *α*_*k*_ is a parameter prior that can be interpreted as belief equivalent to having previously (prior to obtaining *D*) seen *α*_*k*_ individuals that satisfy ***V***_*S*_ = ***v***_*S*_ and *T* = *k*, and (4) *α* = ∑_*k*_ *α*_*k*_. The parameter *α* is called the *prior equivalent sample size* (*pess*) and controls how much smoothing occurs in estimating probability parameters; the higher is *pess*, the greater is the smoothing that occurs [13]. For the DP methods used in this paper, we assume a simple non-informative prior and set *α*_*k*_ = 1/*K* where *K* is the number of values that *T* can take. In the datasets that we use, the target is binary and hence *K* is equal to 2.

#### Search strategy

**Fig 2:**
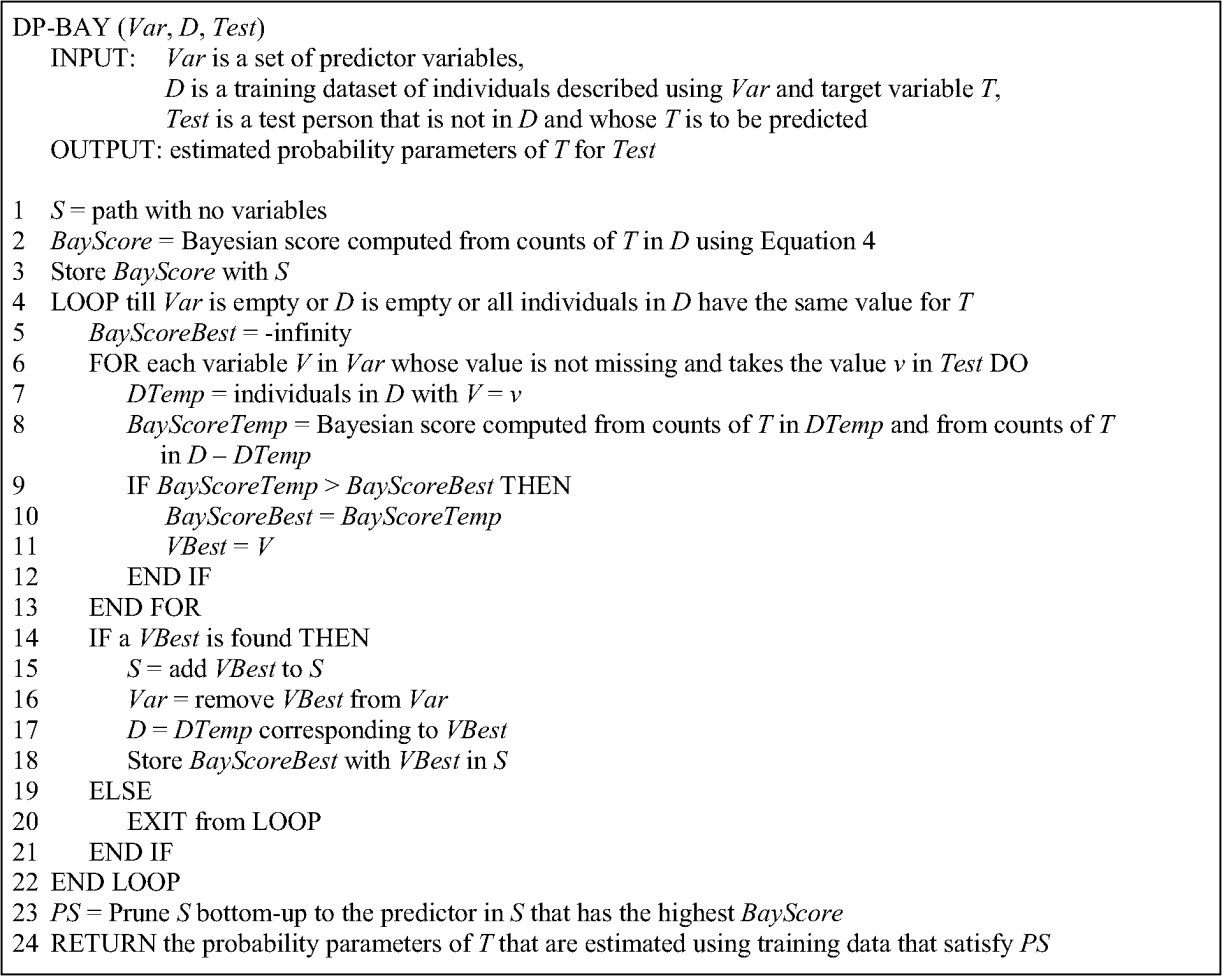
Pseudocode for the DP-BAY method.

The pseudocode for DP-BAY is given in Fig 2. The DP-BAY method uses greedy forward-stepping search to identify features to add to *S*. Given a training dataset and a test person for whom we want to estimate the distribution of *T*, DP-BAY begins with *S* that has no features. It successively adds features to *S* by identifying at each step the best feature to add using a Bayesian score.

At some point in the search, let *M* have the path *S*, let *D* be the set of training individuals that satisfy *S*, and let *V* = *v* in the test person be a candidate predictor variable under consideration. Then, *V* = *v* is temporarily added to the current path *S* to obtain *S’* and the Bayesian score for *S’* is computed from 1) *DTemp* that contains training individuals that satisfy *S’* (line 7 in Fig 2), and 2) *D*–*DTemp* that contains training individuals that satisfy *S* but not *S’*. The details of the Bayesian score are given in the next section. The reason we use *DTemp* and *D*–*DTemp* rather than only *DTemp* to evaluate *V* is because each candidate variable *V* will potentially result in a *DTemp* with a different number of individuals and we wish to evaluate potential variables on the same set of individuals. The growth of the path is terminated when no more features are available, no more training individuals are available, or the remaining individuals all have the same value for *T*.

DP-BAY performs pruning to reduce overfitting as follows. During the growing phase, DP-BAY records the corresponding Bayesian score that is associated with each new feature that is added to *S*. Pruning is performed on *S* bottom-up so that the final feature in *S* is associated with the highest score recorded during the growing phase (see line 23 in Fig 2). The probability parameters of the pruned path *PS* are estimated from the training data that satisfy *PS*, and this model is returned by the method. Variables whose values are missing in the test person are handled naturally by not considering them as candidate variables in line 6 in Fig 2.

#### Bayesian model score

We outline a set of equations that lead up to the Bayesian score that we use to evaluate *S’* which is obtained from path *S* by temporarily adding a candidate variable *V*. We seek to derive the posterior probability *P*(*S*' | *D*) using Bayes theorem as follows:

$$\begin{array}{l} P\left(S\prime |D\right)=P\left(S\prime |DTemp,D-DTemp\right)\\ =\frac{P\left(DTemp,D-DTemp|S\prime \right)P\left(S\prime \right)}{\sum_{S\prime }P\left(DTemp,D-DTemp|S\prime \right)P\left(S\prime \right)}=\frac{P\left(DTemp,D-DTemp|S\prime \right)P\left(S\prime \right)}{P\left(DTemp,D-DTemp\right)}. \end{array}$$ (2)

Since *P*(*D*) = *P*(*DTemp*, *D*−*DTemp*) is a constant, *P*(*S*' | *D*) is proportional to *P*(*DTemp*, *D*−*DTemp* | *S*') × *P*(*S*') where *P*(*S*') is the prior probability of *S'*. We assume that every path *S* is equally likely a priori. Thus, *P*(*S*' | *D*) is proportional to *P*(*S*' | *DTemp*, *D*−*DTemp*), which can be computed in closed form under the assumptions of multinomial sample, parameter independence, and parameter modularity [13]. Given that the prior probabilities of the parameter set follow Dirichlet distributions, the Bayesian score is given by:

$$P\left(DTemp,D-DTemp|S\prime \right)=\prod_{j=1}^{2}\left(\frac{\Gamma \left(\alpha_{j}\right)}{\Gamma \left(N_{j}+\;\alpha_{j}\right)}\times \prod_{k=1}^{K}\frac{\Gamma \left(N_{jk}+\;\alpha_{jk}\right)}{\Gamma \left(\alpha_{jk}\right)}\right).$$ (3)

The symbol Γ represents the gamma function. The variable *j* indexes the two samples *DTemp* and *D-DTemp*. In each sample, the variable *k* indexes the counts for each of the *K* values of *T*. *N*_1*k*_ is the number of individuals in *DTemp* that take the value *k* for *T*. Similarly, *N*_2*k*_ is the number of individuals in *D-DTemp* that take the value *k* for *T*. Here, $N_{j}=\sum_{k=1}^{K}N_{jk}$. The *α*_*jk*_ represent the Dirichlet parameters of the prior probability distribution of the parameters, and $\alpha_{j}=\sum_{k=1}^{K}\alpha_{jk}$.

The terms in Eq (3) can become so small that they cause problems in computing with adequate numerical precision. Thus, it is better to calculate them in logarithmic form, and we define the Bayesian score for a predictor *V* that is under consideration as:

$$BayScore\left(V\right)=\sum_{j=1}^{2}\left(\frac{log\Gamma \left(\alpha_{j}\right)}{\text{log}\;\Gamma \left(N_{j}+\alpha_{j}\right)}+\sum_{k=1}^{K}\frac{\text{log}\;\Gamma \left(N_{jk}+\alpha_{jk}\right)}{\text{log}\;\Gamma \left(\alpha_{jk}\right)}\right).$$ (4)

For the experiments described in this paper, we assume a simple non-informative parameter prior and set *α*_*jk*_ = 1 / *K* where *K* is the number of values that *T* can take. In the datasets that we use, since the target is binary *K* is equal to 2, *α*_*jk*_ is equal to ½, and *α*_*j*_ is equal to 1.

### The DP-IG method

The DP-IG method differs from DP-BAY in that it uses information gain (IG) instead of the Bayesian score. Information gain for a predictor *V* that is under consideration is given by

$$IG\left(V\right)=H\left(D\right)-\frac{\left|DTemp\right|}{\left|D\right|}H\left(DTemp\right)-\frac{\left|D-DTemp\right|}{\left|D\right|}H\left(D-DTemp\right),$$ (5)

where *H*(*E*) denotes entropy of a dataset *E* and is given by the expression

$$H\left(E\right)=-\sum_{k}P\left(T=k\right)log_{2}P\left(T=k\right),$$ (6)

where *P*(*T* = *k*) is the proportion of the dataset *E* that includes samples that have the value *k* for *T*. Thus, the DP-IG method uses Eq (5) instead of Eq (4) in Fig 2. The DP-IG method is similar to LazyDT in that it uses information gain; however, DP-IG differs from LazyDT in that the latter performs pruning and uses the BDeu measure for calculating the probability parameters to prevent overfitting.

### The DP-AUC method

**Fig 3:**
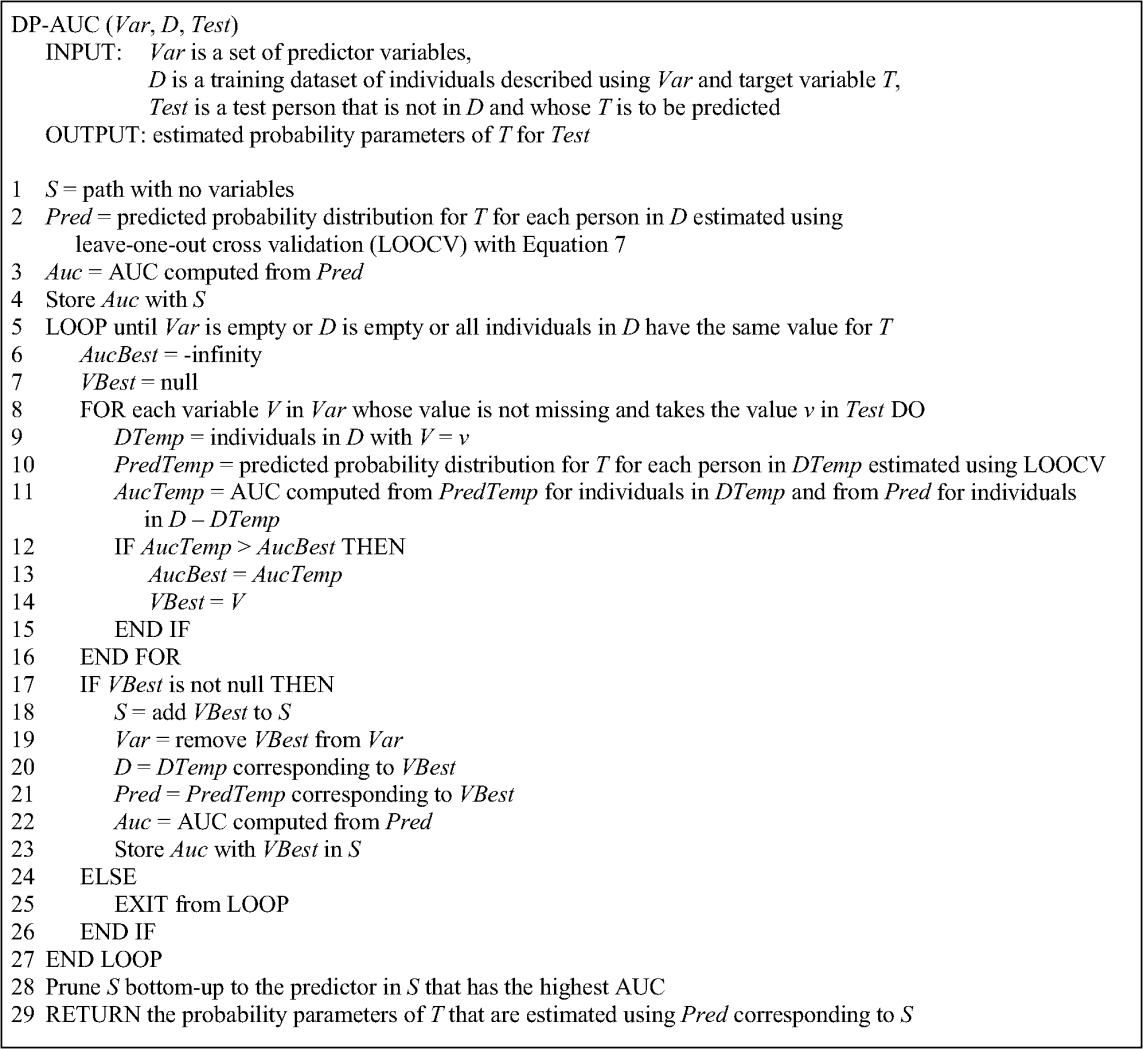
Pseudocode for the DP-AUC method.

The pseudocode for DP-AUC method is given in Fig 3. DP-AUC differs from the other two algorithms in that it uses AUC to evaluate *S’* which is obtained from path *S* by temporarily adding a candidate variable *V*. Computing the AUC requires obtaining a prediction for *T* for each person in the training set of individuals that matches *S*’. This is done using leave-one-out cross validation. Specifically, one of the training individuals that matches *S*’ is left out and Eq (1) is used to estimate the parameters of the distribution over *T* for the left-out person from the remaining training individuals that match *S*’. Using the probability parameters, a probabilistic prediction is obtained for *T* for the left-out person with the following equation:

$$P\left(T=p|V_{S}=v_{S}\right)=\theta_{p},$$ (7)

where *p* denotes the positive value of the target variable and *θ*_*p*_ is the parameter estimate for the positive value. The process is repeated by leaving out each of the training individuals in turn so that a prediction is obtained for each person. Using this leave-on-out method for computing predictions, the AUC for *S’* is computed from the predictions for the individuals in *DTemp* and the individuals in *D*–*DTemp* (see line 11 in Fig 3).

## Experimental Methods

In this section we describe the datasets, the experimental methods, and the performance measures that we used to evaluate the methods.

### Datasets

We used four clinical datasets and one genomic dataset. The clinical datasets included 1) a pneumonia dataset with one outcome, 2) a sepsis dataset with two outcomes that are labelled sepsis-d and sepsis-s (see Table 1), 3) a heart failure dataset with two outcomes that are labeled heart failure-d and heart failure-c (see Table 1), and 4) a heparin induced thrombocytopenia (HIT) dataset with one outcome. The genomic dataset is on Alzheimer’s disease. All of the datasets were collected in other studies, and made available for this study by the researchers who had originally collected the data. Waiver of consent was obtained, and patient records/information was anonymized and de-identified prior to analysis. The University of Pittsburgh Institutional Review Board approved this study. We provide brief descriptions of these datasets below (see Table 1).

#### Pneumonia dataset

Community acquired pneumonia is one of the most common infectious diseases and is an important cause of mortality and morbidity worldwide. The pneumonia dataset that we used contains several hundred clinical, laboratory, and radiographic variables on 2,287 adult patients who were admitted to a hospital with a diagnosis of community acquired pneumonia. The data was collected by the Pneumonia Patient Outcomes Research Team (PORT) in three geographical locations including Pittsburgh, Boston, and Halifax, Nova Scotia [14]. A variety of clinical data was collected at the time of presentation and several outcomes at 30 days were assessed. One key goal of the PORT project was to develop accurate criteria for prognosis of patients with pneumonia that could provide guidance on which patients should be hospitalized and which patients might be safely treated at home.

The binary outcome variable is called dire outcome. A patient was considered to have experienced a dire outcome if death occurred within 30 days of presentation or one or more specific, severe complications occurred during the hospitalization.

#### Sepsis dataset

Sepsis is a syndrome of systemic inflammation in response to infection that can result in multi-system organ dysfunction and failure. The sepsis dataset that we used contains 21 variables as predictors that included demographic, clinical, inflammatory markers, and genetic variables on 1,673 patients who were admitted to a hospital with a diagnosis community acquired pneumonia. The data was collected in the GenIMS (Genetic and Inflammatory Markers of Sepsis) project from patients presenting to the emergency departments of 28 hospitals in western Pennsylvania, Connecticut, Michigan, and Tennessee in the United States [15].

Two binary outcome variables, which were the focus of investigation in the original study, were selected for prediction: (1) the occurrence of death within 90 days of enrollment in the study, and (2) the development of severe sepsis during hospitalization.

#### Heart failure dataset

Heart failure affects 5 million people in the United States leading to about one million hospital admissions each year. The heart failure data we used was collected by 192 general acute care hospitals in Pennsylvania in the year 1999 and consists of heart failure patients who were hospitalized from the Emergency Departments. The dataset consists of 11,178 patients and 21 predictor variables that including demographic, clinical, laboratory, electrocardiographic, and radiographic findings[16].

Two binary outcome variables were selected for prediction: (1) the occurrence of death from any cause during the hospitalization, and (2) the development of one or more serious medical complications (including death) during the hospitalization.

#### Heparin induced thrombocytopenia (HIT) dataset

HIT is a life-threatening adverse event that may occur after exposure to heparin and is characterized by a greater than 50% fall in platelet count and is often associated with new thrombosis, typically occurring 5–14 days after the start of heparin. This dataset consists of 549 post-surgical cardiac patient cases who received heparin after surgery. The patient cases were represented by 59 features that were likely to be predictive of HIT. The binary outcome variable is the development of HIT, which was assessed by expert reviewers[17].

#### Alzheimer’s disease genomic dataset

Alzheimer’s disease is a neurodegenerative disease and is the most common cause of dementia associated with aging. The predominant form of the disease is called late-onset Alzheimer’s disease (LOAD) in which the age of onset of symptoms is typically after 65 years. The dataset that we used comes from a genome-wide association study in which genotype data were collected on 1411 individuals, of which 861 had LOAD and 550 did not [18]. For each individual, the genotype data consists of 312,318 single nucleotide polymorphisms (SNPs) obtained after applying quality controls. We applied chi square to rank SNPs that are predictive of development of LOAD and used in our experiments the genotype data on the top ranked 100 SNPs. The binary outcome variable is development of LOAD.

### Experiments

We implemented the DP methods in MATLAB (version R2012a). For the CART method we used the ClassificationTree implementation in MATLAB (version R2012a) with the modification that the predicted probabilities were estimated from counts in leaf nodes using Eq 1 which uses smoothing since this smoothing is used by all the DP methods. We applied the DP-AUC, DP-IG, DP-BAY, and CART methods to predict the outcome variables in the datasets that are shown in Table 1. We set the value of the *pess* parameter to 1 in Eq 1. In the CART method we set the splitting criterion to “deviance” which uses information gain to select predictors to include in the decision tree. Continuous variables were discretized using the entropy-based method developed by Fayyad and Irani [19].

We evaluated the methods using 20-fold cross-validation. Each dataset was randomly partitioned into 20 approximately equal sets, such that each set had a similar proportion of individuals who developed the positive outcome. For each method, we combined 19 sets and evaluated it on the remaining test set, and we repeated this process once for each possible test set. We thus obtained a prediction for the outcome variable for every individual in a dataset. The AUC and Brier-skill-score results reported in the next section are based on these predictions.

We ran all four methods on a PC with a two 1.80 GHz Intel Xeon processors and 32 GB of RAM, and running the 64-bit Windows 7 operating system.

### Performance measures

We evaluated the performance of the methods with AUC, which is a measure of discrimination and evaluates how well a method differentiates between a positive and negative outcome. We also evaluated the quality of the predictions with the Brier score and the Brier-skill score[20]. The Brier score (BS) is a widely used to assess the quality of probabilistic predictions of binary outcomes. BS is negatively oriented, assigning lower values to better predictions, and can take values between 0 and 1. We report 1 –BS rather than BS so that the score is positively oriented. The Brier skill score (BSS) compares a model’s predictions to the predictions of a reference model such as a model that predicts with the prevalence of the positive outcome in the data. BSS is positively oriented, assigning higher values to better predictions, and can take values between negative infinity and 1. A BSS that is greater than 0 indicates that a model’s predictions are better than the reference predictions and a BSS smaller than 0 indicates the opposite. Finally, for each of the DP methods, we examined the proportion of individuals for which the decision-path model produced a different prediction compared to the CART model.

## Results

### AUC results

Table 2 gives the AUCs for the four methods on seven outcomes in the five datasets. The results show that the highest average AUC is obtained by DP-BAY (mean = 0.748, 95% C.I. (0.704, 0.792)) with DP-IG close behind. Table 3 shows results from pair-wise comparisons from a two-sided paired-samples t-test of the AUC performance of the methods. DP-BAY and DP-IG have statistically similar performance and both have statistically significantly better performance than either DP-AUC or CART at the 5% significance level.

**Table 2:** AUCs for the datasets and outcomes shown in Table 1.

Dataset	CART	DP-AUC	DP-IG	DP-BAY
pneumonia	0.659 (0.611, 0.707)	0.654 (0.626, 0.683)	0.732 (0.699, 0.765)	**0.788** (0.766, 0.811)
sepsis-d	0.669 (0.620, 0.718)	0.746 (0.715, 0.776)	**0.766** (0.738, 0.795)	0.757 (0.723, 0.792)
sepsis-s	0.658 (0.629, 0.687)	0.670 (0.644, 0.696)	0.710 (0.688, 0.732)	**0.716** (0.691, 0.742)
heart failure-d	0.682 (0.653, 0.710)	0.707 (0.683, 0.730)	0.734 (0.708, 0.761)	**0.751** (0.731, 0.770)
heart failure-c	0.653 (0.636, 0.670)	0.644 (0.630, 0.658)	0.699 (0.687, 0.712)	**0.712** (0.700, 0.725)
HIT	0.818 (0.771, 0.864)	0.847 (0.811, 0.883)	0.830 (0.790, 0.870)	**0.849** (0.811, 0.888)
Alzheimer	0.624 (0.589, 0.658)	0.598 (0.575, 0.621)	0.650 (0.648, 0.692)	**0.676** (0.654, 0.698)
Mean	0.680 (0.634, 0.727)	0.693 (0.629, 0.756)	0.734 (0.694, 0.773)	**0.748** (0.704, 0.792)

For each method the table gives the mean AUC obtained from 20-fold cross-validation and 95% confidence intervals. The last row gives the mean AUC and 95% confidence intervals over all datasets. Highest mean AUC for each outcome is in bold.

**Table 3:** Two-sided paired-samples t test comparing the pairwise performance of the four methods on AUC.

	CART	DP-AUC	DP-IG	DP-BAY
CART		-0.851 (0.427)	-5.290 (**0.002)**	-5.460 (**0.002)**
DP-AUC			-3.090 (**0.022)**	-3.260 (**0.017)**
DP-IG				-1.860 (0.113)

In each cell in the table, the number on top is the mean difference between the method in the row and the method in the column and the number at the bottom is the corresponding p value. The mean difference is negative when method in the row has a lower AUC than the method in the column. Results in bold indicate p values of 0.05 or smaller.

### BS and BSS results

Table 4 provides the 1—BS and BSS results for the four methods on seven outcomes. The highest average 1—BS and the highest average BSS are obtained by DP-BAY (mean = 0.80 for 1—BS, mean = 0.15 for BSS) followed by DP-IG, DP-AUC, and CART in that order. Table 5 shows results from pair-wise comparisons from a two-sided paired-samples t-test of the BSS performance of the methods. DP-BAY and DP-IG have statistically similar performance and both have statistically significantly better performance than either DP-AUC or CART at the 5% significance level. Overall, the relative performances on the BSS by the four methods are similar to their relative performance on the AUC.

**Table 4:** BS and BSS for the datasets and outcomes shown in Table 1.

Dataset	CART	DP-AUC	DP-IG	DP-BAY
pneumonia	0.76 / 0.09	0.77 / 0.10	0.80 / 0.15	**0.86 / 0.20**
sepsis-d	0.79 / 0.11	0.81 / 0.13	**0.84 / 0.17**	0.82 / 0.15
sepsis-s	0.70 / 0.06	0.71 / 0.08	0.74 / 0.10	**0.75 / 0.11**
heart failure-d	0.73 / 0.08	0.74 / 0.08	0.79 / 0.11	**0.81 / 0.13**
heart failure-c	0.67 / 0.02	0.66 / 0.02	0.71 / 0.06	**0.83 / 0.07**
HIT	0.84 / 0.16	0.86 / 0.19	0.86 / 0.19	**0.88 / 0.22**
Alzheimer	0.62 / 0.07	0.61 / 0.05	0.66 / 0.12	**0.68 / 0.14**
Mean	0.73 / 0.08	0.74 / 0.09	0.77 / 0.13	**0.80 / 0.14**

For each method the table gives the mean 1—BS and BSS obtained from 20-fold cross-validation. The last row gives the mean 1—BS and BSS over all datasets. Highest mean 1—BS and BSS for each outcome are in bold.

**Table 5:** Two-sided paired-samples t test comparing the pairwise performance of the four methods on BSS.

	CART	DP-AUC	DP-IG	DP-BAY
CART		-0.851 (0.427)	-5.290 (**0.002)**	-5.460 (**0.002)**
DP-AUC			-3.090 (**0.022)**	-3.260 (**0.017)**
DP-IG				-1.860 (0.113)

In each cell in the table, the number on top is the mean difference between the method in the row and the method in the column and the number at the bottom is the corresponding p value. The mean difference is negative when method in the row has a lower BSS than the method in the column. Results in bold indicate p values of 0.05 or smaller.

### Differences in predictions between DP and CART methods

For each of the three DP methods, Table 6 shows the proportion of individuals for each of the datasets for which the DP method’s prediction differed from CART’s prediction. DP-IG, which uses an information theoretic criterion, had the smallest proportion of individuals where its predictions differed from that of CART (mean = 0.16). DP-AUC had the largest proportion of individuals where its predictions differed from that of CART (mean = 0.25) and the proportion for DP-BAY was between that of DP-IG and DP-AUC (mean = 0.19).

**Table 6:** Proportion of test individuals for which the decision-path model is different from the path in CART model.

Dataset	DP-AUC	DP-IG	DP-BAY
pneumonia	0.31	0.17	0.23
sepsis-d	0.29	0.15	0.16
sepsis-s	0.24	0.18	0.20
heart failure-d	0.19	0.17	0.16
heart failure-c	0.26	0.18	0.19
HIT	0.21	0.12	0.14
Alzheimer	0.25	0.18	0.22
Mean	0.25	0.16	0.18

For each method the table gives the mean proportion obtained from 20-fold cross-validation. The last row gives the mean proportion over all datasets.

## Discussion

The personalized decision-path methods are similar to instance-based methods. Instance-based methods store the training samples, identify a subgroup from the training samples when presented with a test sample, and use the subgroup to derive a prediction. The subgroup is identified using a distance metric that computes the distance between every training sample and the test sample based on the features in the test sample. The canonical instance-based method is the *k* nearest-neighbor (*k*NN) technique. Given a test sample, *k*NN selects the *k* most similar training samples, and returns the majority vote of the target values for the *k* training instances [21]. In a similar fashion the decision-path methods identify a subgroup of the training samples that have the same features as the test sample; they differ from *k*NN in that only a subset of the features is used in determining the subgroup. It is important to note that instance-based and decision-path methods both use knowledge of features of the test sample that is being predicted but do not use information from other test samples in the test set that would lead to contamination during learning from knowledge of features in the test set samples.

The results show that two of the three personalized decision path methods, namely, DP-BAY and DP-IG performed significantly better than CART and DP-AUC in terms of both AUC and Brier scores. DP-BAY performed slightly better than DP-IG but not statistically significantly so. DP-AUC did not perform as well as the other DP methods even though it was designed to optimize AUC directly which was one of the performance measures.

Personalized models like decision-path models can suffer from overfitting when model parameters are estimated from small samples, and mechanisms to prevent overfitting are necessary. Pruning is one way to prevent overfitting and is used in all three DP methods. In addition, the Bayesian score used in DP-BAY averages over parameter values and is another way to prevent overfitting. The information gain score has properties similar to the Bayesian score and thus its performance is similar to DP-BAY. Part of the reason for the poor performance of DP-AUC may be that the AUC estimates can be poor for small sample sizes that occur with long paths with many predictors. The AUC estimate is not penalized for smaller sample sizes as is the Bayesian and the information gain score.

The DP methods that are described in this paper have several limitations. One limitation is that they handle only discrete variables and continuous variables have to be discretized. Another limitation is that the methods use greedy search and performance may improve with more sophisticated search strategies, such as best-first search. Another limitation is using decision trees with conjunction as the only way to combine features. In future work, we plan to investigate search strategies beyond greedy search, and go beyond conjunction to disjunction of conjunctions as a richer way of combining features. We also plan to explore the use of model averaging where we average the predictions of several good models and compare the performance to that of model selection where we select a single good model as we have done with the current DP methods.

The datasets used in our experiments were collected in experimental studies where features were measured in a standardized way on all samples. Additional evaluation of DP methods is needed to assess their generalizability to observational data that are available from electronic health record (EHR) systems where feature measurements may be impacted by changing processes of measurement and recording. Also, further study is needed to evaluate the portability of such methods from one institution to other institutions with similar and different EHR systems.

## Conclusion

We introduced three new personalized predictive methods that derive decision-path models, and evaluated them on several medical datasets to predict clinical outcomes. Based on the results of AUC and Brier scores, DP-BAY and DP-IG had excellent comparative performance. These results provide support for including these DP methods to predict clinical outcomes in biomedical data. More generally, the results reported here provide support for additional investigation of model-based personalized methods for use in predicting clinical and biomedical outcomes.
